# The Stress and Adversity Inventory for Adults (Adult STRAIN) in German: An overview and initial validation

**DOI:** 10.1371/journal.pone.0216419

**Published:** 2019-05-09

**Authors:** Sarah C. Sturmbauer, Grant S. Shields, Eva-Luca Hetzel, Nicolas Rohleder, George M. Slavich

**Affiliations:** 1 Department of Psychology, Chair of Health Psychology, Friedrich-Alexander-Universität Erlangen-Nürnberg, Germany; 2 Center for Neuroscience and Department of Psychology, University of California, Davis, CA, United States of America; 3 Cousins Center for Psychoneuroimmunology and Department of Psychiatry and Biobehavioral Sciences, University of California, Los Angeles, CA, United States of America; Chiba Daigaku, JAPAN

## Abstract

Life stress is a key determinant of poor mental and physical health, but until recently no instrument existed for efficiently assessing cumulative stress exposure and severity across the entire lifespan. The Stress and Adversity Inventory (STRAIN) is an online, interview-based stress assessment system that was developed to address this need. We examined the concurrent, predictive, and discriminant validity of a German translation of the STRAIN by administering the instrument, along with several other measures of stress and health, to 298 adults (81 men, 217 women, *M*_age_ = 30.3 years). The German STRAIN demonstrated excellent concurrent validity, as evidenced by associations with other instruments assessing early adversity (|*r*s|≥.62, *p*s≤.001). It also correlated with instruments assessing recent life event exposure in adulthood (|*r*s|≥.48, *p*s≤.001), as well as recent perceived stress (|*r*s|≥ .25, *p*s≤.001) and recent chronic stress levels (|*r*s|≥ .19, *p*s≤.001). Additionally, the German STRAIN showed strong predictive validity in relation to anxiety symptoms (|*r*s|≥ .22, *p*s≤.001) and depressive symptoms (|*r*s|≥ .33, *p*s≤.001). Finally, the German STRAIN showed good discriminant validity, with lifetime stressor count being unrelated to personality features like neuroticism. These results demonstrate that the German version of the STRAIN is a valid tool for assessing lifetime stress exposure and severity. Additional research is needed to examine how the German STRAIN predicts psychological and biological stress reactivity and physical health outcomes.

## Introduction

Life stress contributes to a wide variety of serious mental and physical health problems that cause substantial morbidity and mortality. Starting early in life, for example, maternal psychosocial stress exposure prospectively predicts offspring’s symptoms of anxiety and depression in childhood and adolescence [1]. Childhood adversity also confers increased vulnerability to adulthood stress exposure [2] and promotes risk for mental health disorders [3]. Additionally, stressors occurring during childhood and adolescence predict increased *allostatic load*, characterized as dysregulation in multiple biological systems that underlie health [4]. Collectively, these findings support the possibility that stressors occurring during early development enhance vulnerability to biological risk factors for poor lifespan health [2, 4].

Stress exposure occurring during adulthood also can greatly impact mental health by precipitating the development of anxiety disorders and depression [5]. Moreover, findings from both population-based and clinical studies indicate that uncontrollable life events and chronic stressors are associated with increased body weight, which is a strong risk factor for cardiovascular disease (CVD) [6]. Life stress exposure in adulthood also increases risk for somatic and physical disorders including asthma, certain cancers, and neurodegenerative disorders, all of which can greatly deteriorate lifespan health [7, 8]. Perhaps most importantly, stress promotes premature biological aging and has been shown to predict early mortality [9]. Considered together, these effects demonstrate that stress occurring during both childhood and adulthood affect lifespan health and disease risk. Presently, however, the stress assessment instruments used do not permit inferences about the *cumulative effects* of stress exposure on health because no instruments have existed for systematically assessing stressors occurring over the entire lifespan.

This lack of empirical research directly relating to *lifespan* stress exposure and health is striking given that many theoretical models have proposed that stressors occurring over the entire life course may exert a *cumulative effect* on biobehavioral pathways that in turn increase risk for disease [10, 11]. For example, it has been suggested that acute and chronic stressors occurring over the life course may influence the activity and interplay of the hypothalamic-pituitary-adrenal (HPA) axis and autonomic nervous system (ANS) over time, which in turn promotes inflammatory processes that have a direct effect on cumulative disease risk [12–15]. Again, however, very few studies have actually assessed all of the acute and chronic stressors that people have experienced in order to directly test these models.

### Measurement of life stress

Given the absence of an instrument for assessing lifetime stress exposure, investigators have resorted to using a variety of mesures that assess exposure to stress during specific developmental periods. For example, prenatal stress has been assessed using mothers’ self-reported stressors through all stages of pregnancy. Similarly, childhood maltreatment and adversity have been measured using retrospective self-report questionnaires or interviews, such as the Childhood Trauma Questionnaire [16] and Adverse Childhood Experience Questionnaire [17]. Although easy to administer, these instruments only assess certain stressors in early life (e.g., early abuse, neglect), leaving all other stressors—and the rest of the person’s life—unmeasured. Additionally, these instruments do not quantify the precise timing or duration of each stressor experienced, which prevents investigators from comparing the effects of acute versus chronic stressors or those occurring during specific periods of early development.

In contrast, stress exposure occurring during adulthood has been most commonly assessed using self-report checklist measures, such as the Social Readjustment Rating Scale (SRRS; [18]) and the Life Events Checklist for DSM-V (CES-D; [19]). The Trier Inventory for Chronic Stress (TICS; [20]) has been commonly used to assess chronic stress levels over the past three months in various domains. The Perceived Stress Scale (PSS) is perhaps the most frequently used instrument for assessing overall perceived stress burden occurring over the past four weeks [21]. The Life Events and Difficulties Schedule and UCLA Life Stress Inventory are well-validated interview-based systems for assessing adulthood stress exposure, but given the substantial cost and time associated with these instruments, they are rarely used [22].

As alluded to above, one of the main issues here is that these existing measures of early life and adulthood stress do not map well onto the theoretical models described above. More specifically, whereas most contemporary theoretical models employ a cumulative lifespan approach, the instruments that are most frequently used for assessing stress exposure focus only on specific periods of a person’s life. As a result, much of the data that presently exist on life stress and health do not directly address the life course theories they aim to test.

### Stress and Adversity Inventory

The Stress and Adversity Inventory for Adults (Adult STRAIN) was designed in the U.S. to address these limitations by providing investigators with an easy-to-use online interviewing platform for assessing stress exposure occurring across the entire life course (http://www.strainsetup.com; [23]). To accomplish this, the STRAIN combines the simplicity of a self-report instrument with the sophistication of a structured interview for assessing life stress. Like interview-based measures, for example, the STRAIN assesses the severity, frequency, timing, and duration of each stressor that is endorsed. Questions are written colloquially and appear one-by-one, making them easy to answer. The Adult STRAIN takes about 18 minutes to complete and assesses 55 different major life stressors–including 26 acute life events and 29 chronic difficulties–that span 12 major life domains (e.g., housing, work, financial, marital/partner relationship) and 5 social-psychological characteristics (e.g., interpersonal loss, physical danger, humiliation). Based on the data collected, more than 445 raw variables are generated that can be combined into 115 different stress exposure scores. In turn, analyses can be based on type of exposure outcome (e.g., lifetime stressor count vs. severity), type of stressors experienced (e.g., acute life events vs. chronic difficulties), timing of exposure (e.g., early life vs. adulthood life stress, or continuous by age), primary life domain of the exposures, and their core social-psychological characteristics.

### Present study

In the present study, we first created a German version of the Adult STRAIN by forward-translating and then back-translating the instrument according to established procedures. Here, we report on the two main lifetime stress exposure outcomes generated by the STRAIN—namely, the total count and total cumulative severity of all stressors experienced over the lifespan. Next, we tested the concurrent validity of the STRAIN against commonly used instruments for assessing stress in different stages of life. Namely, we compared the STRAIN with German versions of the Adverse Childhood Experience Questionnaire (ACE; [17, 24]), Childhood Trauma Questionnaire–Short Form (CTQ-SF; [16, 25]), Life Event Checklist for DSM-V (LEC-5; [19, 26]), Perceived Stress Scale (PSS; [21, 27]), and Trier Inventory for Chronic Stress (TICS; [20]). To examine the predictive validity of the STRAIN, we examined associations between the STRAIN and measures of trait anxiety and depressive symptoms—specifically, German versions of the State-Trait Anxiety Inventory (STAI; [28, 29]), Center for Epidemiological Studies Depression Scale (ADS-L; [30, 31]), and Brief Patient Health Questionnaire (PHQ-D; [32, 33]). To examine the STRAIN’s discriminant validity, we compared the STRAIN with the Big Five personality traits [34, 35] using the German version of the Ten Item Personality Inventory (TIPI-G; [36]). Based on prior research [23], we expected the German version of the STRAIN to demonstrate good usability, concurrent validity, predictive validity, and discriminant validity.

## Method

### Participants and procedure

Participants were recruited from the local community (Erlangen, Germany) from January 2017 to May 2017. The study was online and took approximately 90 minutes to complete. After providing written informed consent, participants were directed to three separate online modules, each covering one topic (i.e., health, mood, and stress) with various questionnaires (e.g., health status, demographic factors, anxiety, depressive symptoms, and stress exposure) and the STRAIN. All participants who completed all three assessments were included in analyses, producing a final sample of 298 adults (81 men, 217 women) with a mean age of 30.3 years old (*SD* = 12.9 years old; range: 18–80).

Level of education was relatively high, with 42% having completed the German “Abitur” (equivalent to high school diploma), 21% with a Bachelor’s degree, and 17% with a Master’s degree. 93% of all participants identified their race as “White”. Regarding mental health, 40 participants reported being currently diagnosed with a mental health disorder (13.4%) and 30 participants reported using psychotropic drugs (10.1%). The study protocol was approved by the ethics committee of the Friedrich-Alexander-University Erlangen-Nürnberg and was carried out in accordance with the declaration of Helsinki. Psychology students enrolled at the Friedrich-Alexander-University Erlangen-Nürnberg received course credit for participating in the study.

### Measures

#### Lifetime stress exposure

Lifetime stress exposure was assessed using the German version of the Adult STRAIN. The interview was forward translated from English to German and subsequently back translated by two independent bilingual speakers. Content-related inconsistencies were then discussed within the research group and the best fitting wording was selected. Similar to the original English version, the final German version consists of 55 core stressors and the optional Transition to College (TTC) module. For each stressor that was endorsed, a series of tailored follow-up questions were prompted to assess the severity, frequency, timing, and duration of each reported stressor. This information was then combined to generate different lifetime stress exposure summary scores for each participant [23]. Here, we report on the two main lifetime stress exposure outcomes generated by the STRAIN—namely, the total count and total cumulative severity of all stressors experienced over the lifespan.

#### Early adversity

To assess traumatic experiences occurring before eighteen years old, the ACE was used [24]. It consists of 10 items (e.g., “Were your parents separated or divorced?”) assessing adverse experiences during childhood and adolescent. Each item allows forced choice ratings (*yes* vs. *no*) on various dimensions including abuse and neglect.

Childhood adversity was also assessed using the CTQ-SF [25]. The short form includes 25 items on early adversity (e.g., “I had to wear dirty clothes”) and assesses neglect and abuse, resulting in five dimensions of childhood maltreatment. Responses ranged from 1 (*never true*) to 5 (*very often true*), and were averaged to create an overall score with higher scores indicating more early adversity. For the primary analyses, the sum score of all traumatic childhood experiences was used. Internal consistency was excellent, α = .91.

#### Life events

To assess potential traumatic life events, we used the Life Event Checklist for DSM-5 (LEC-5; [26]), which is a 16-item self-report instrument to screens for 16 events (e.g., “Flood”) known to potentially result in PTSD or psychological distress. For each stressor, respondents can choose between “happened to me”, “witnessed it”, “learned about it”, “part of my job”, “not sure” or “doesn’t apply”. Life events that were scored as “happened to me” were then summed to create an overall index for experienced amount of life events.

#### Perceived stress

Participants’ levels of perceived stress over the past four weeks were assessed using the 10-item version of the Perceived Stress Scale (PSS; [27]). For example, participants reported how “uncontrollable” or “unpredictable” they regarded their lives on a five-point Likert scale ranging from 0 (*never*) to 4 (*very often*). The resulting score represents a person’s overall perceived stress level, with higher scores indicating greater perceived burden. Internal consistency was very good, α = .89.

#### Chronic difficulties

To measure participants’ chronic stress exposure over the past three months, we used the Trier Inventory for Chronic Stress (TICS; [20]). This questionnaire assesses nine domains (e.g. Work Overload: “I have too many tasks to perform.”). Participants provided responses on 57 items on a five-point Likert scale respect to how often they had a certain situation or experience. Internal consistency was excellent, α = 0.95.

#### Anxiety and depressive symptoms

Participants’ anxiety levels were assessed with the STAI [28]. The inventory consists 40 items (e.g., Trait: “I make decisions easily.”, State: “I am tense.”) that assess state (current state) and trait (in general) anxiety. Items are rated on a four-point Likert scale. Internal consistency for the STAI State and Trait scales were α = .57 and α = .94, respectively.

Depressive symptoms occurring over the past week were assessed using the German version of the CES-D (ADS-L; [30]), which consists 20 items (e.g. “I felt depressed.”) and allows ratings on a four-point Likert scale. Internal consistency was excellent, α = .92. Depressive symptoms over the past two weeks were also assessed using the PHQ-D [32], which contains 9 items and uses a four-point Likert scale. An example would be: “Feeling down, depressed or hopeless”. Internal consistency was very good, α = .88.

#### Personality traits

Participants’ Big Five personality traits (i.e., openness to experience, conscientiousness, extraversion, agreeableness, neuroticism) were assessed using the TIPI-G [36], which includes 10 items (e.g., “I see myself as someone who is generally trusting”). The TIPI-G correlates strongly with longer measures, such as the Big Five Inventory [36]. Internal consistency was good, ranging from α = .33 to α = .74.

### Data analyses

All analyses were conducted in R (v. 3.4.0) and RStudio (v. 1.0.143). Normal distributions of variables were calculated with the Shapiro-Wilk test. To analyze the STRAIN’s validity, multiple regression models were run. All multiple regression models included the following covariates: age, sex, self-reported mental health diagnosis, and self-reported psychotropic drug use. Outliers were detected by considering cook’s distance (values >1), leverage (cutoff value .2), and studentized residuals (cutoff value ± 3). Cook’s distance and leverage values were satisfying. Therefore, outliers were only excluded when studentized residuals were greater than ± 3. All regression models were calculated both including and excluding outliers, with both sets of results reported below.

## Results

### Usability and acceptability

The median time to complete the German version of the Adult STRAIN was 24 minutes and 51 seconds (interquartile range = 19 minutes 32 seconds– 33 minutes 1 second). Overall acceptability of the instrument was excellent, with no participants terminating the interview and no reported complaints or psychosocial distress as a result of answering the questions.

### Descriptive statistics for lifetime stress exposure

On average, participants reported 15.65 stressors over the life course (*SD* = 10.61; range 0–71; possible range 0–166). The overall lifetime severity of these stressors was 37.61 (*SD* = 26.80; range 0–163; possible range 0–265). Adjusting for age, these totals were significantly lower than the total lifetime stressor count (F(2,500 = 53.86, p < .001) and total lifetime stressor severity (F(2,500) = 65.15, p < .001) obtained for the English STRAIN. However, participants in the original English validation study were significantly older (nearly 8 years on average) than those in the present study (German sample: *M*_*age*_ = 30.3, *SD*_*age*_ = 12.9; English sample: *M*_*age*_ = 37.82, *SD*_*age*_ = 11.72; F(1,501 = 44.73, *p* < .001), which may help explain this difference.

**Fig 1:**
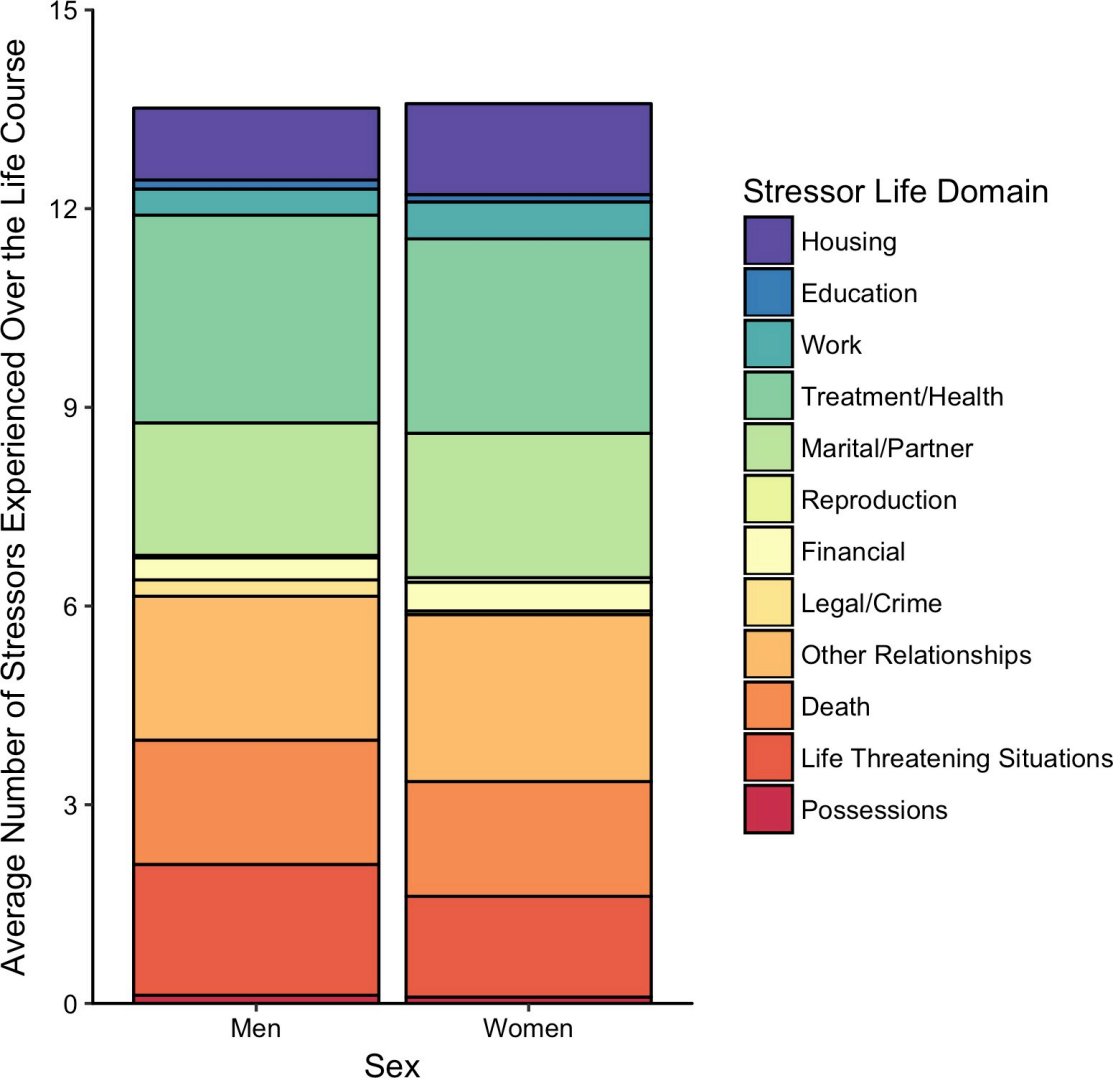
Lifetime stressor count by stressor category for men (*n* = 81) and women (*n* = 217). Stressor Life Domains: Men reported more legal/crime stressors than women (*p* = .005).

**Fig 2:**
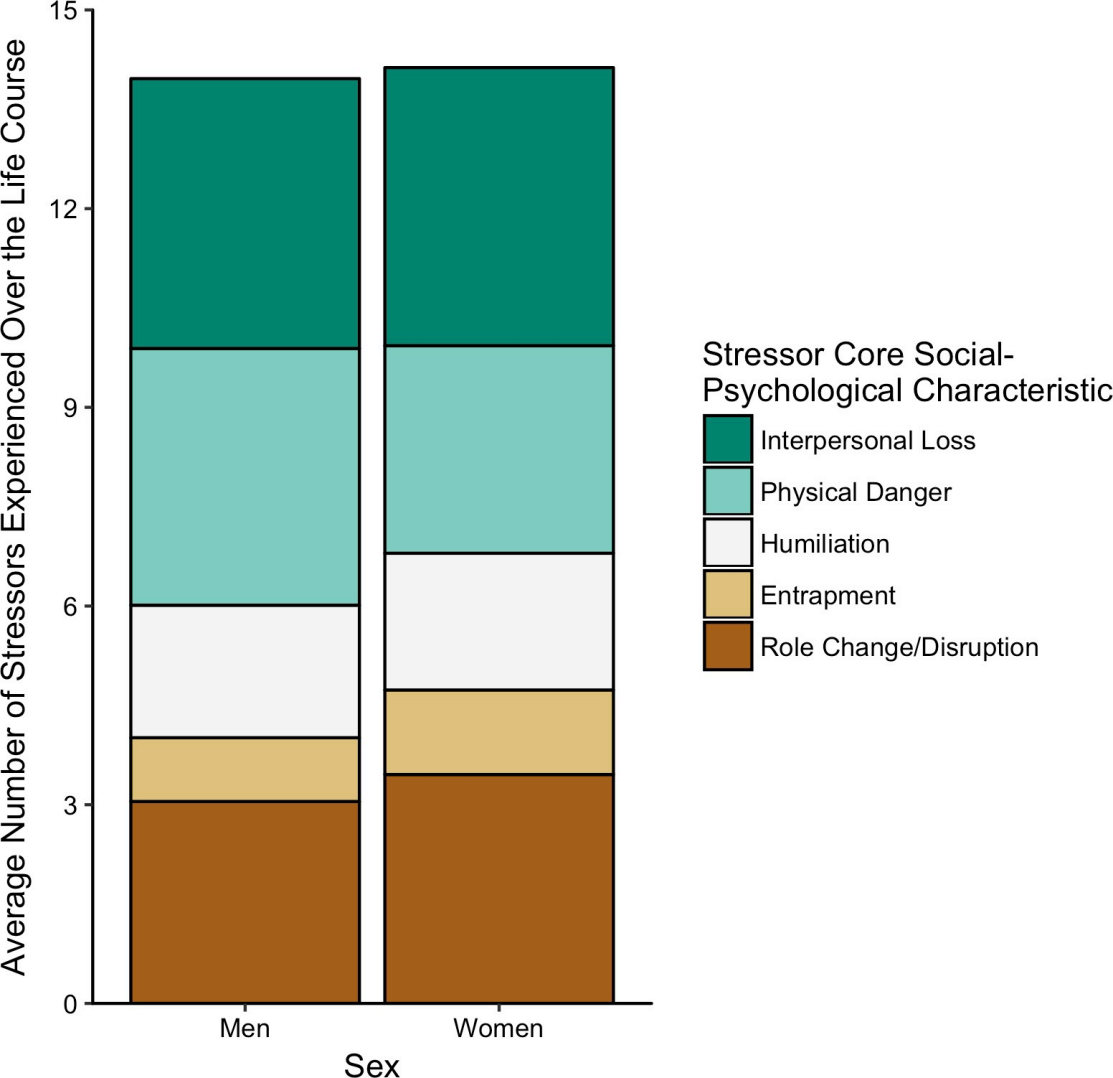
Lifetime stressor count by core social-psychological characteristics for men (*n* = 81) and women (*n* = 217). Stressor Core Social-Psychological Characteristics: Women experienced more entrapment stressors than men (*p* = .038).

Regarding sex and race, on average, men and women did not differ in the number of lifetime stressors they experienced (*t*(296) = -.315, *p* = .753). Similarly, lifetime stressor count did not vary by race (*F*(4, 293) = 1.23, *p* = .296), though with the sample being 93% White, we were underpowered to detect racial differences in stress exposure. As expected, we found that older individuals reported more lifetime stressors (*r* = .35, *p* < .001) and greater lifetime stressor severity (r = .32, p < .001). Welch’s *t*-tests revealed that individuals with a self-reported diagnosed mental health disorder reported more lifetime stressors (*t*(46.2) = -5.25, *p* < .001) and greater lifetime stressor severity (*t*(46.8) = -6.23, *p* < .001). Looking more closely at the stress exposure categories, as depicted in Fig 1, we found that men experienced more legal/crime stressors than women (*p* = .005). For the core social-psychological characteristics, as depicted in Fig 2, we found that women experienced more entrapment stressors than men (*p* = .038).

### Latent structure of lifetime stressor data

**Fig 3:**
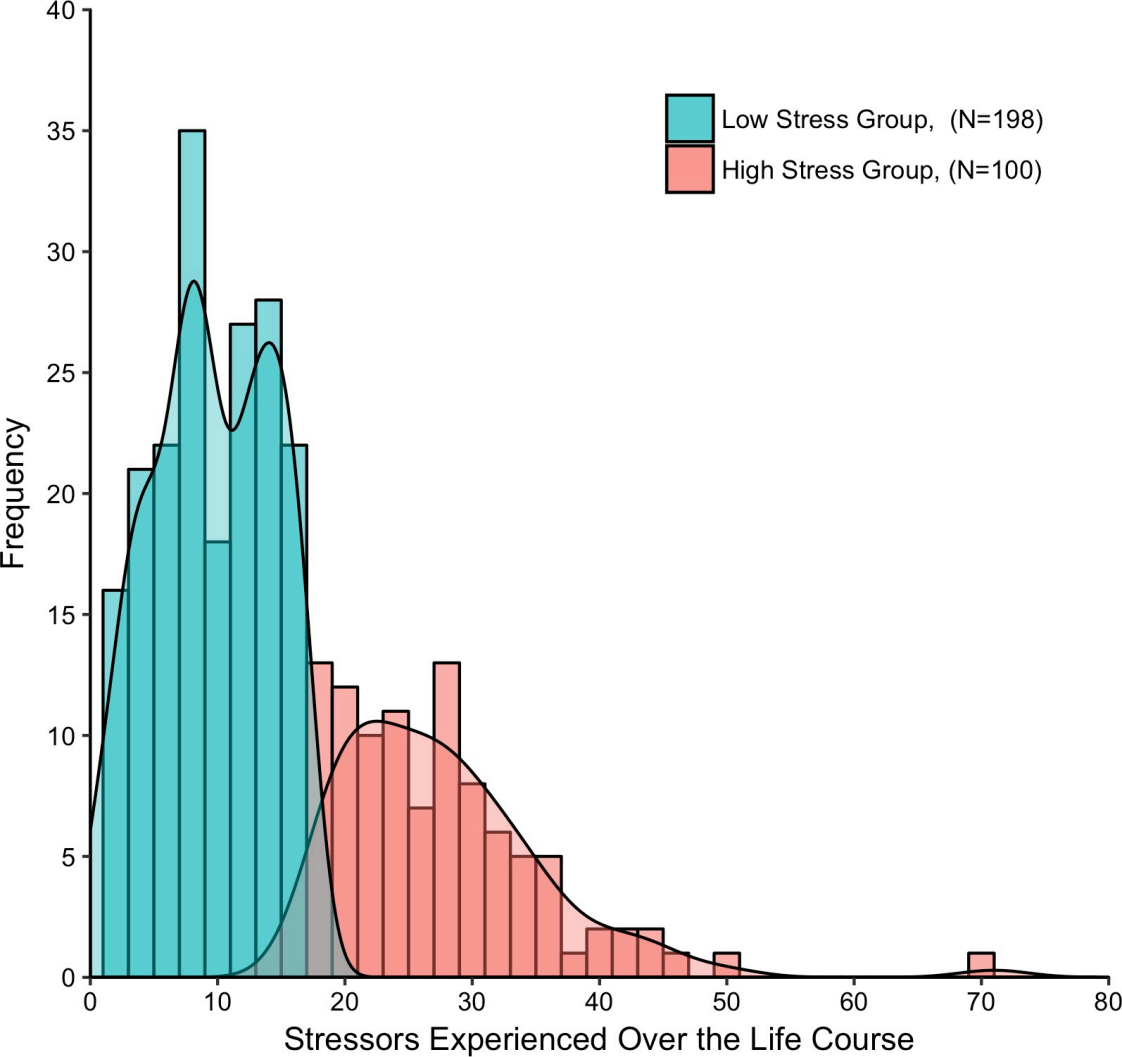
Latent structure of the lifetime stressor data. Two latent classes best fit the underlying distribution of the overall lifetime stressor count data—namely, a low-stress group (*n* = 198; total lifetime stressors: *M* = 9.52, *SD* = 4.69) and a high-stress group (*n* = 100; total lifetime stressors: *M* = 27.79, *SD* = 8.38).

The underlying distribution of overall lifetime stressor count was assessed using a latent class analysis, testing the fit of 1–9 latent classes both assuming equal variance and not. Equivalent to the English STRAIN, we found that two latent classes with unequal variance best fit the data (Fig 3)—namely, a low-stress group (*n* = 198; total lifetime stressor count: *M* = 9.52, *SD* = 4.69) and a high-stress group (*n* = 100; total lifetime stressor count: *M* = 27.79, *SD* = 8.38).

### Concurrent validity

In terms of validity, we first examined the concurrent validity of the STRAIN against other commonly used scales for assessing life stress. Multiple separate regression models were calculated to examine the extent to which the CTQ-SF, ACE, LEC-5, PSS, and TICS predicted the STRAIN’s main indices of lifetime stressor count and cumulative severity. All multiple regression models controlled for age, sex, self-reported mental health diagnosis, and self-reported psychotropic drug use.

#### Early adversity

Concurrent validity analyses for early adversity compared the main STRAIN indices with those derived from the ACE and CTQ-SF. As expected, both lifetime stressor count and total lifetime stress exposure severity were strongly correlated with participants’ total ACE score (Count: *r* = .62, *p* < .001; Severity: *r* = .62, *p* < .001) and total CTQ-SF score (Count: *r* = .64, *p* < .001; Severity: *r* = .62, *p* < .001). Comparing the correlations among scores derived from these instruments, we found no differences between the strength of association between the STRAIN and the ACE, and the STRAIN and the CTQ-SF (Count: one-tailed z-difference = -0.404, *p* = .343; Severity: one-tailed z-difference = -0.059, *p* = .476).

Parallel analyses were conducted while controlling for covariates. In these analyses, total lifetime stressor count as assessed by the STRAIN remained significantly associated with participants’ self-reported number of adverse childhood experiences assessed by the ACE (β = 0.56; Δ*R*^2^ = .282, *p* < .001). The model parameters were nearly identical after excluding four outliers (*p* < .001; calculation of outliers was model-based and is described in the Method). Similarly, total lifetime stressor severity as assessed by the STRAIN remained significantly associated with the number of adverse childhood experiences assessed by the ACE (β = .54; Δ*R*^2^ = .260, *p* < .001). Again, the model parameters were nearly identical after excluding three outliers (*p* < .001).

All correlations between the STRAIN and the subscales of the CTQ-SF are shown in Table 1. As shown, we found the strongest association between the STRAIN indices and the Emotional Abuse subscale. Controlling for covariates, total lifetime stressor count was still significantly associated with participants’ reported number of traumatic childhood experiences (β = .55; Δ*R*^2^ = .257, *p* < .001). After excluding four outliers, the model parameters were nearly identical (*p* < .001). Similarly, total lifetime stressor severity was significantly associated with participants’ total CTQ-SF score (β = .51; Δ*R*^2^ = .223, *p* < .001). Again, after excluding four outliers, the model parameters were nearly identical (*p* < .001).

**Table 1:** Zero-order correlations between the STRAIN indices and the subscales of the Childhood Trauma Questionnaire (CTQ-SF).

		*M*	*SD*	1	2	3	4	5	6	7	8
1	STRAIN Lifetime Stressor Count	15.65	10.61	–	.93***	.59***	.42***	.40***	.63***	.42***	-.32***
2	STRAIN Lifetime Stressor Severity	37.61	26.80		–	.57***	.39***	.36***	.61***	.43***	-.30***
3	CTQ-SF Emotional Neglect	10.07	4.58			–	.43***	.46***	.75***	.68***	-.51***
4	CTQ-SF Sexual Abuse	5.71	2.39				–	.53***	.43***	.44***	-.13***
5	CTQ-SF Physical Abuse	5.68	1.66					–	.51***	.41***	-.16***
6	CTQ-SF Emotional Abuse	8.50	4.24						–	.55***	-.38***
7	CTQ-SF Physical Neglect	7.05	2.75							–	-.27***
8	CTQ-SF Trivialize	.58	.96								–

*M* = mean*; SD* = standard deviation

Total *N* = 298

*** *p* < .001

#### Life events

As expected, both of the STRAIN’s main lifetime stress exposure indices correlated strongly with participants’ life event stress, as assessed by the LEC-5 (Count: *r* = .50, *p* < .001; Severity: *r* = .48, *p* < .001). Controlling for covariates did not affect these results, as total lifetime stressor count was still significantly associated with participants’ total LEC-5 score (β = 0.43; Δ*R*^2^ = .175, *p* < .001). Moreover, the model parameters were nearly identical after excluding four outliers (*p* < .001). Total lifetime stressor severity as assessed by the STRAIN was also significantly associated with participants’ total LEC-5 score after controlling for covariates (β = 0.41; Δ*R*^2^ = .158, *p* < .001). Again, the model parameters were nearly identical after excluding four outliers (*p* < .001).

#### Perceived stress and chronic difficulties

Total lifetime stressor count and severity were both strongly correlated with participants’ total PSS score (Count: *r* = .25, *p* < .001; Severity: *r* = .32, *p* < .001). These associations were not affected by controlling for covariates, as total lifetime stressor count was still significantly associated with participants’ total PSS score in this fully adjusted model (β = .20; Δ*R*^2^ = .035, *p* < .001). Moreover, the model parameters were nearly identical after excluding two outliers (*p* < .001). Similarly, total lifetime stressor severity as assessed by the STRAIN remained significantly associated with participants’ total amount of perceived stress over the last four weeks in the fully adjusted model (β = 0.26; Δ*R*^2^ = .059, *p* < .001). Again, the model parameters were nearly identical after excluding three outliers (p < .001).

We then compared the STRAIN with the TICS, a commonly used instrument for assessing chronic stress over the past three months. Correlations between the STRAIN and the subscales of the TICS are shown in Table 2. In unadjusted bivariate associations, the STRAIN indices correlated significantly with all subscales of the TICS (|*r*s|≥.19, *p*s≤.001).

**Table 2:** Zero-order correlations between the STRAIN indices and the subscales of the Trier Inventory of Chronic Stress (TICS).

		*M*	*SD*	1	2	3	4	5	6	7	8	9	10	11	12
1	STRAIN Lifetime Stressor Count	15.65	10.61	–	.93****	.25***	.37***	.26***	.20***	.23***	.29***	.31***	.24***	.19***	.38***
2	STRAIN Lifetime Stressor Severity	37.61	26.80		–	.29***	.43***	.26***	.24***	.28***	.33***	.34***	.28***	.25***	.43***
3	TICS Work Overload	21.87	6.56			–	.45***	.50***	.30***	.59***	.48***	.41***	.27***	.47***	.67***
4	TICS Social Overload	14.29	5.20				–	.56***	.15***	.28***	.44***	.39***	.12***	.21***	.55***
5	TICS Pressure to Perform	24.35	6.43					–	.23***	.40***	.45***	.42***	.19***	.30***	.61***
6	TICS Work Discontent	19.32	6.15						–	.51***	.47***	.34***	.47***	.50***	.62***
7	TICS Excessive Demands from Work	12.58	4.69							–	.51***	.47***	.43***	.67***	.67***
8	TICS Lack of social Recognition	9.47	3.63								–	.45***	.30***	.36***	.65***
9	TICS Social Tensions	12.03	4.23									–	.28***	.39***	.63***
10	TICS Social Isolations	14.07	5.38										–	.41***	.54***
11	TICS Chronic Worrying	10.59	3.95											–	0.62***
12	TICS Screening Scale	30.19	6.71												–

*M* = mean*; SD* = standard deviation

Total *N* = 298

*** *p* < .001

Total lifetime stressor count as assessed by the STRAIN was still significantly associated with participants’ reported amount of chronic stress as assessed by the TICS screening scale after controlling for covariates (β = .32; Δ*R*^2^ = .093, *p* < .001). Moreover, the model parameters were nearly identical after excluding three outliers (*p* < .001). Similar associations were found for participants’ total lifetime stressor severity as assessed by the STRAIN and their recent chronic stress levels after controlling for covariates (β = .36; Δ*R*^2^ = .059, *p* < .001). Again, the model parameters were nearly identical after excluding two outliers (*p* < .001).

Comparing both measures, we found that the STRAIN was more strongly associated with the TICS as compared to the PSS. This was true for total lifetime stressor count as assessed by the STRAIN (one-tailed z-difference = -1.747, *p* = .04), but not for total lifetime stressor severity (one-tailed z-difference = -1.563, *p* = .059). Comparing the PSS and the TICS with the LEC-5, we found smaller associations of the PSS with the STRAIN than for the LEC-5 with the STRAIN (Count: one-tailed z-difference = -3.566, *p* = .0002; Severity: one-tailed z-difference = -2.222, *p* = .013). Regarding chronic difficulties, we also found smaller associations of the TICS with the STRAIN than the LEC-5. This applied only for total lifetime stressor count (one-tailed z-difference = -1.819, *p* = .043) and not for total lifetime stressor severity (one-tailed z-difference = -0.659, *p* = .255).

#### Summary

To summarize, the STRAIN demonstrated excellent concurrent validity, as evidenced by strong associations between the STRAIN and several of the most commonly used instruments for assessing stress levels during different time periods. More specifically, the STRAIN was strongly associated with instruments assessing exposure to both adverse childhood experiences and traumatic life events, and these effects were robust to adjustment for both covariates and outliers. Similar results were found for adulthood life stress. Here, the STRAIN correlated strongly with instruments assessing recent life event exposure, recent perceived stress, and recent chronic stress levels, with some evidence that the STRAIN was more strongly associated with the LEC-5 than the other two measures. Details of these regression models are provided in Table 3 and Table 4.

**Table 3:** Multiple regression models for the convergent validity for STRAIN Lifetime Stressor Count.

	STRAIN Lifetime Stressor Count				
Model	Adj. R^2^	Δ R^2^	*F*	*p*	*SE*
Covariates	.20		19.7	< .001	9.48
Covariates + ACE	.48	.282	56.92	< .001	7.61
Covariates	.20		19.7	< .001	9.48
Covariates + CTQ-SF	.46	.257	51.47	< .001	7.80
Covariates	.20		19.7	< .001	9.48
Covariates + LEC-5	.38	.175	36.86	< .001	8.38
Covariates	.20		19.7	< .001	9.48
Covariates + PSS	.23	.035	19.12	< .001	9.29
Covariates	.20		19.7	< .001	9.48
Covariates + TICS Screening Scale	.29	.093	25.62	< .001	8.92

Covariates: age, sex, self-reported mental health disorder and self-reported intake of psychotropic drugs.

ACE = Adverse Childhood Experience Questionnaire; CTQ-SF = Childhood Trauma Questionnaire–Short Form; LEC-5 = Life Event Checklist for DSM-5; PSS = Perceived Stress Scale; TICS = Trier Inventory of Chronic Stress screening scale; SE = Standard Residual Error.

**Table 4:** Multiple regression models for the convergent validity for STRAIN Lifetime Stressor Severity.

	STRAIN Lifetime Stressor Severity				
Model	Adj. R^2^	Δ R^2^	*F*	*p*	*SE*
Covariates	.22		22.54	< .001	23.59
Covariates + ACE	.49	.260	57.19	< .001	19.21
Covariates	.22		22.54	< .001	23.59
Covariates + CTQ-SF	.45	.223	49.32	< .001	19.9
Covariates	.22		22.54	< .001	23.59
Covariates + LEC-5	.37	.158	34.66	< .001	21.06
Covariates	.22		22.54	< .001	23.59
Covariates + PSS	.28	.059	24.26	< .001	22.72
Covariates	.22		22.54	< .001	23.59
Covariates + TICS Screening Scale	.34	.059	31.73	< .001	21.75

Covariates: age, sex, self-reported mental health disorder and self-reported intake of psychotropic drugs.

ACE = Adverse Childhood Experience Questionnaire; CTQ-SF = Childhood Trauma Questionnaire–Short Form; LEC-5 = Life Event Checklist for DSM-5; PSS = Perceived Stress Scale; TICS = Trier Inventory of Chronic Stress screening scale; SE = Standard Residual Error.

### Predictive validity

Next, we assessed the predictive validity of the STRAIN by examining the extent to which it predicted participants’ anxiety and depressive symptoms over the past two weeks. Scores on the two subscales of the STAI (trait and state) were highly correlated (*r* = .67, *p* < .001), so separate multiple regression models were calculated.

#### Trait anxiety

The STRAIN was significantly associated with participants’ trait anxiety levels, as assessed by the STAI Trait scale (Count: *r* = .22, *p* < .001; Severity: *r* = .27, *p* < .001). Controlling for covariates did not affect these results, as total lifetime stressor count remained significantly associated with participants’ trait anxiety levels in adjusted analyses (β = .17; Δ*R*^2^ = .022, *p* = .004). Moreover, the model parameters were nearly identical after excluding one outlier (*p* = .002). Similar results were found for models assessing lifetime stressor severity, wherein the STRAIN still significantly predicted participants’ trait anxiety levels as assessed by the STAI, even after adjusting for covariates (β = .21; Δ*R*^2^ = .034, *p* < .001). Again, the model parameters were nearly identical after excluding one outlier (*p* < .001).

#### State anxiety

Likewise, the STRAIN was significantly associated with participants’ state anxiety levels, as assessed by the STAI State scale (Count: *r* = .26, *p* < .001; Severity: *r* = .28, *p* < .001). Controlling for covariates did not affect these results, as total lifetime stressor count remained significantly associated with participants’ state anxiety levels in adjusted analyses (β = .26; Δ*R*^2^ = .052, *p* < .001). Moreover, the model parameters were nearly identical after excluding two outliers (p < .001). Similar results were found in controlled analyses examining the association between total lifetime stressor severity and participants’ state anxiety levels (β = .21; Δ*R*^2^ = .057, *p* < .001). Again, the model parameters were nearly identical after excluding two outliers (*p* < .001).

#### Depressive symptoms

We conducted parallel analyses for depressive symptoms, which examined how the STRAIN associated with two commonly used instruments for assessing depressive symptoms—namely, the German version of the CES-D Scale (ADS-L), which assesses symptoms occurring over the past week, and the PHQ-D, which assesses symptoms over the past two weeks. Scores on these two depression scales were highly correlated (*r* = .80, *p* < .001), so separate multiple regression models were calculated.

The STRAIN was significantly associated with participants’ depressive symptom levels as assessed by the ADS-L (Count: *r* = .26, *p* < .001; Severity: *r* = .31, *p* < .001). Controlling for covariates did not affect these results, as total lifetime stressor count remained strongly associated with participants’ depressive symptom levels as assessed by the ADS-L (β = .24; Δ*R*^2^ = .046, *p* < .001). The model parameters were nearly identical after excluding two outliers (*p* < .001). Similar results were obtained for total lifetime stressor severity, which also strongly predicted participants’ depressive symptoms levels as assessed by the ADS-L in these adjusted analyses (β = .29; Δ*R*^2^ = .064, *p* < .001). Again, excluding two outliers produced model parameters that were nearly identical (*p* < .001).

Results using participants’ scores on the PHQ-D were highly convergent, showing significant associations between the STRAIN and depressive symptom levels as assessed by the PHQ-D (Count: *r* = .33, *p* < .001; Severity: *r* = .37, *p* < .001). Again, controlling for covariates did not affect these results, as total lifetime stressor count remained strongly associated with participants’ total PHQ-D scores (β = .32; Δ*R*^2^ = .080, *p* < .001). Moreover, the model parameters were nearly identical after excluding one outlier (*p* < .001). Similar results were obtained for total lifetime stressor severity, which was also strongly associated with participants’ depressive symptom levels as assessed by the PHQ-D in these adjusted analyses (β = .35; Δ*R*^2^ = .095, *p* < .001). As before, the model parameters were nearly identical after excluding three outliers (*p* < .001).

#### Summary

To summarize, the STRAIN demonstrated excellent predictive validity, as evidenced by its strong associations with participants’ trait anxiety levels, state anxiety levels, and depressive symptom levels as assessed by two different instruments. Details of these regression models are provided in Table 5 and Table 6.

**Table 5:** Multiple regression model parameters for the predictive validity of the STRAIN with the Subscales of the STAI.

	Anxiety Levels Assessed by the STAI									
Model	STAI Trait Anxiety					STAI State Anxiety				
	Adj. R^2^	Δ R^2^	*SE*	*F*	*p*	Adj. R^2^	Δ R^2^	*SE*	*F*	*p*
Covariates	.21		9.02	20.39	< .001	.10		9.90	9.07	< .001
Covariates + STRAIN Lifetime Stressor Count	.23	.022	8.91	18.39	< .001	.15	.052	9.62	11.29	< .001
Covariates	.21		9.02	20.39	< .001	.10		9.90	9.07	< .001
Covariates + STRAIN Lifetime Stressor Severity	.24	.034	8.84	19.7	< .001	.15	.057	9.59	11.7	< .001

Covariates: age, sex, self-reported mental health disorder and self-reported intake of psychotropic drugs.

STAI = State-Trait Anxiety Inventory; SE = Standard Residual Error.

**Table 6:** Multiple regression model parameters for the predictive validity of the STRAIN with participant’s depressive symptom levels.

	Current Depressive Symptom Levels									
Model	ADS-L					PHQ-D				
	Adj. R^2^	Δ R^2^	*SE*	*F*	*p*	Adj. R^2^	Δ R^2^	*SE*	*F*	*p*
Covariates	.14		9.73	12.72	< .001	.16		4.82	14.54	< .001
Covariates + STRAIN Lifetime Stressor Count	.18	.046	9.48	14.05	< .001	.23	.080	4.59	18.95	< .001
Covariates	.14		9.73	12.72	< .001	.16		4.82	14.54	< .001
Covariates + Lifetime Stressor Severity	.20	.064	9.38	15.74	< .001	.25	.096	4.54	20.55	< .001

Covariates: age, sex, self-reported mental health disorder and self-reported intake of psychotropic drugs.

ADS-L = Center for Epidemiological Studies Depression Scale; PHQ-D = Brief Patient Health Questionnaire; SE = Standard Residual Error.

### Discriminant validity

Finally, we assessed the discriminant validity of the STRAIN by examining its association with the TIPI-G [36]. Similar to the English STRAIN [23], lifetime stressor count as assessed by the German STRAIN was weakly correlated with openness to experience (*r* = .16, *p* = .005). Similar results were found for total lifetime stressor severity, which was weakly correlated with both openness to experience (*r* = .12, *p* = .033) and neuroticism (*r* = -.13, *p* = .026). No significant associations were found for extraversion, agreeableness, or conscientiousness (|*r*s|<-.03, *p*s>.136). To test whether these results were robust to statistical adjustment, we reran these analyses while controlling for age, sex, self-reported mental health diagnosis, and self-reported psychotropic drug use. Total lifetime stressor count remained significantly associated with openness to experience (β = .12; Δ*R*^2^ = 0.014, *p* = .018), but this association was no longer significant after excluding two outliers (β = .01, *p* = .058). Total lifetime stressor severity, in turn, was no longer associated with openness to experience (β = .08; Δ*R*^2^ = -0.006, *p* = .107) or with neuroticism (β = -.04; Δ*R*^2^ = 0.001, *p* = .119) in these adjusted analyses, and excluding three outliers did not affect these model parameters (*p*s>.05). In sum, German STRAIN stressor count was weakly correlated with openness to experience, and stressor severity was weakly correlated with both openness to experience and neuroticism, but these associations were not robust to adjustment for covariates or outliers.

In comparison, the ACE significantly correlated with agreeableness with and without adjusting for covariates and outliers (β = -.17; Δ*R*^2^ = 0.03, *p* = .003), but it was not related to any of the other personality traits with or without statistical adjustment (|*r*s|≤.05, |βs|≤.10, *p*s ≥ .10). The CTQ-SF, in turn, was only associated with conscientiousness when adjusting for covariates and outliers (β = -.17; Δ*R*^2^ = 0.028, *p* = .003) and was not related to any of the other personality traits, with or without statistical adjustment (|*r*s|≤ .05, |βs|≤.10, *p*s≥.10). The LEC-5 was only correlated with openness to experience (β = -.24; Δ*R*^2^ = 0.054, p = .0502), and this association attenuated when excluding outliers (*p* = .031). It was not related to any of the other Big Five personality traits with or without statistical adjustment (|*r*s|≤.05, |βs|≤.10, *p*s≥.10). With or without adjustment for covariates, the PSS was significantly associated with extraversion (β = -.18; Δ*R*^2^ = 0.033, *p* < .001), neuroticism (β = -.51; Δ*R*^2^ = 0.214, *p* < .001), conscientiousness (β = -.24; Δ*R*^2^ = 0.054, *p* < .001), openness to experience (β = -.22; Δ*R*^2^ = 0.046, *p* < .001), and agreeableness (β = -.18; Δ*R*^2^ = 0.031, *p* < .001). Excluding outliers did not affect these model parameters (*p*s < .001). The TICS screening scale was significantly associated with extraversion (β = -.14; Δ*R*^2^ = 0.020, *p* = .009), neuroticism (β = -.29; Δ*R*^2^ = 0.070, *p* < .001), openness to experience (β = -.16; Δ*R*^2^ = 0.026, *p* = .003), and agreeableness (β = -.21; Δ*R*^2^ = 0.044, *p* < .001) when adjusting for covariates. Excluding outliers did not affect these associations (*p*s < .001). Finally, the TICS significantly correlated with conscientiousness (β = -.12; Δ*R*^2^ = 0.054, *p* = .035), but after excluding outliers, this association was no longer significant (*p* = .08). All unadjusted analyses are presented in Table 7.

**Table 7:** Zero-order correlations between of all of the stress measures and big five personality traits.

		*M*	*SD*	1	2	3	4	5	6	7	8	9	10	11	11
1	STRAIN Lifetime Stressor Count	15.65	10.61	–	.93**	.62**	.64**	.50**	.25**	.38**	-.06	-.03	-.03	-.08	.16**
2	STRAIN Lifetime Stressor Severity	37.61	26.80		–	.62**	.62**	.48**	.32**	.43**	-.09	-.04	-.04	-.13*	.12*
3	ACE	1.43	1.87			–	.73**	.39**	.19**	.33**	.002	-.13**	-.03	-.09	.09
4	CTQ-SF	37.60	12.29				–	.44**	.21**	.33**	-.08	-.09	-.09	-.09	.05
5	LEC-5	1.50	1.70					–	.06	.11	.03	.03	.06	.03	.13*
6	PSS	2.65	.72						–	.63**	-.21	-.16**	-.28**	-.58**	-.22**
7	TICS Screening Scale	30.19	6.72							–	-.18**	-.19	-.15**	-.39**	-.16**
8	TIPI-G: Extraversion	8.56	2.74								–	-.04	.05	.24**	.31**
9	TIPI-G: Agreeableness	10.12	2.07									–	.19**	.09	.12*
10	TIPI-G: Conscientiousness	10.91	2.16										–	.20**	.002
11	TIPI-G: Neuroticism	9.07	2.69											–	.30**
12	TIPI-G: Openness to Experience	10.24	2.08												–

*M* = mean*; SD* = standard deviation; ACE = Adverse Childhood Experience Questionnaire; CTQ-SF = Childhood Trauma Questionnaire–Short Form; LEC-5 = Life Event Checklist for DSM-5; PSS = Perceived Stress Scale; TICS = Trier Inventory of Chronic Stress screening scale; TIPI-G = Ten Item Personality Inventory.

Total *N* = 298

*p < .05

**p < .01

## Discussion

Although life stress contributes to a wide variety of serious mental and physical health problems, very few published studies have measured cumulative stress exposure occurring over the entire life course [22, 37]. The Adult STRAIN addresses this issue by providing an easy-to-use, online interview-based platform for assessing individuals’ total exposure to stress over the lifespan, and it does so by measuring the severity, frequency, timing, and duration of each stressor experienced [23]. The aim of the present study was to validate a translation of the Adult STRAIN into the German language and to confirm its usability and acceptance. Then, we tested the instrument’s concurrent, predictive, and discriminant validity.

In the present sample of adults recruited from a German population, participants completed the STRAIN in approximately 25 minutes. No complaints or distress resulted from answering the questions, thus demonstrating excellent overall acceptance. On average, participants reported approximately 16 stressors over the life course, which was significantly less than the English validation study sample [23]. Moreover, the STRAIN demonstrated excellent concurrent validity. For example, it was strongly associated with instruments assessing exposure to both adverse childhood experiences and traumatic life events. Above the covariates, almost one third of the variance in the STRAIN’s main indices of lifetime stressor count and cumulative severity was additionally explained by childhood adversity, underscoring the importance of these early development phases, which could enhance the vulnerability to biological risk factors for poor health and stress exposures in adult life [2, 4].

Similar results were found for adulthood life stress. Here, the STRAIN correlated strongly with instruments assessing recent life event exposure, recent perceived stress [21, 27], and recent chronic stress levels. These associations were robust to adjustment for both covariates and outliers, demonstrating excellent concurrent validity of the STRAIN. In addition, total lifetime stressor count as well as severity significantly predicted participants’ trait and state anxiety levels. Similarly, total lifetime stressor count and severity were each associated with more self-reported depressive symptoms [30–33], demonstrating the excellent predictive validity of the STRAIN.

Finally, we assessed the discriminant validity of the STRAIN by examining its association with different personality traits. Similar to the English STRAIN [23], the German STRAIN was not associated with any personality traits after adjusting for covariates and removing outliers. Therefore, the STRAIN’s primary stress exposure indices appear to be unaffected by personality characteristics. When we compared our findings with the English validation study, we found that the underlying distribution of overall lifetime stressor counts was equally distributed between the German population sample and the English validation sample. Due to higher scores in the English sample, however, the means of the total number of stressors reported across the samples differed (high-stress group: 41 vs. 28 stressors, low stress-group: 14 vs. 9 stressors). Further studies using the German population are necessary to examine whether these differences would remain in a more diverse or older sample. When we compared the concurrent validity across the two studies, we found similar results for the Childhood Trauma Questionnaire and the Perceived Stress scale between the English and the German sample. The differences between the zero-order correlation across the two samples were not statistically significant. In addition, because the present study did not include an assessment of social desirability, further research is needed to compare how the STRAIN is related to this construct across the two countries [38–41].

In both samples, lifetime stressor count and severity were not associated with participants’ Big Five personality traits. One main difference between the present sample and the English validation sample is the assessment of predictive validity. The initial validation of the German STRAIN focuses on current anxiety and depressive symptoms, whereas the English validation also assessed executive function, sleep quality, and doctor-diagnosed health problems and autoimmune disorders. Overall, our results are highly consistent with those obtained with the original Stress and Adversity Inventory for Adults (Adult STRAIN) and show that the STRAIN is a valid tool for assessing lifetime stress exposure and severity in various settings.

Although the need for a better stress assessment is universal, this need is particularly critical in Germany. Indeed, recent reports by the Federal Statistical Office (Bundesamt für Statistik) as well as health insurance companies [42–44] have indicated serious increases in perceived stress in the population, as well as increases in hospitalization rates due to depression. Particularly alarming is the increased number of children being hospitalized due to an ICD-10 (F30-F39) diagnosis [45]. Germany has also shown a dramatic increase in suicide rates, with more than 10,000 suicides registered by the Federal Statistical Office in 2015 [46]. According to the World Health Organization, the suicidal rate in Europe is even higher than the worldwide rate [47]. To counter this development, the Adult STRAIN in German could be used to help identify individuals at high risk for poor mental and physical health outcomes [1, 3], and to help advance prevention programs aimed at reducing stress and improving wellbeing in this population.

The present study has some limitations. First, due to cross-sectional design and homogeneous sample, no causal interpretations of the results or generalizations to other ethnic/racial groups can be made and we cannot report on re-test reliability. Second, although adjusted analyses indicated that the lifetime stressor reports were unrelated to personality, un-measured self-reporting biases could still have influenced the results. Third, gender was not equally distributed in our sample. Finally, given that this study utilized self-reported health outcomes, additional research is needed to examine how the German STRAIN predicts clinician-rated measures of mental and physical health, as well as other relevant health outcomes and behaviors [6, 48]. Relatedly, additional research is needed to examine the extent to which the German STRAIN predicts health-relevant biomarkers that cannot be influenced by self-reporting biases, such as HPA axis activation in laboratory settings or diurnal cortisol levels throughout the day. The strength of the STRAIN’s predictive validity could also be expanded by examining its association with other health-relevant biological processes (e.g., ANS and HPA axis activation, cytokine levels, allostatic load, etc.).

Given these limitations, next steps for validating the Stress and Adversity Inventory in German should include a broader validation study that considers HPA axis regulation and inflammatory markers. More diverse samples as well as clinical samples should be used to increase our knowledge of lifetime stress across various groups. Finally, given that early adversity and higher rates of depression are evident in adolescence, there is also a pressing need to translate the Stress and Adversity Inventory for Adolescents (Adolescent STRAIN; [49, 50]) into German in order to examine lifetime stress exposure in this population.

In conclusion, the present data suggest that the German Adult STRAIN assesses lifetime stress exposure in a user-friendly and highly acceptable manner. Moreover, the instrument demonstrates excellent concurrent, predictive, and discriminant validity. We thus conclude that this newly developed German version of the STRAIN can be used by investigators and clinicians working with German-speaking populations in order to assess their lifetime stress exposure (e.g., for research and/or case conceptualization and treatment planning purposes).

## Supporting information

S1 File Data. Dataset including variables on which the current study is based.
(CSV)

S2 File Code book. Code Book of all variables used in the dataset.
(XLSX)

## Abbreviations

ACE: Adverse Childhood Experience Questionnaire
ADS-L: the German version of the Center for Epidemiological Studies Depression Scale
CTQ-SF: Childhood Trauma Questionnaire–Short Form
LEC-5: Life Event Checklist for DSM-5
PHQ-D: Brief Patient Health Questionnaire
PSS: Perceived Stress Scale
SE: Standard Residual Error
STAI: State-Trait Anxiety Inventory
STRAIN: Stress and Adversity Inventory for Adults
TICS: Trier Inventory of Chronic Stress screening scale
TIPI-G: Ten Item Personality Inventory—German
